# Can the implementation of clinical practice guidelines improve clinical competence of physicians and kidney function of patients with type 2 diabetes mellitus?

**DOI:** 10.3389/fmed.2022.977937

**Published:** 2022-12-15

**Authors:** Petra Martínez-Martínez, Alfonso M. Cueto-Manzano, Laura Cortés-Sanabria, Héctor R. Martínez-Ramírez, Enrique Rojas-Campos, Aurora Hernández-Herrera

**Affiliations:** ^1^Departamento de Nefrología, Hospital de Especialidades, Centro Médico Nacional de Occidente, Instituto Mexicano del Seguro Social (IMSS), Guadalajara, Jalisco, Mexico; ^2^Unidad de Investigación Médica en Enfermedades Renales, Hospital de Especialidades, Centro Médico Nacional de Occidente, Instituto Mexicano del Seguro Social (IMSS), Guadalajara, Jalisco, Mexico; ^3^Unidad de Medicina Familiar No. 3, Instituto Mexicano del Seguro Social (IMSS), Guadalajara, Jalisco, Mexico

**Keywords:** educative intervention, primary healthcare, diabetes mellitus, chronic kidney disease, clinical practical guidelines

## Abstract

**Background:**

There are many clinical practice guidelines (CPGs) in Nephrology; however, there is no evidence that their availability has improved the clinical competence of physicians or the outcome of patients with chronic kidney disease (CKD). This study was aimed to evaluate the effect of implementation of CPGs for early CKD on family physicians (FP) clinical competence and subsequently on kidney function preservation of type 2 diabetes mellitus (DM2) patients at a primary healthcare setting.

**Methods:**

A prospective educative intervention (40-h) based on *CPGs for Prevention, Diagnosis and Treatment of Early CKD* was applied to FP; a questionnaire to evaluate clinical competence was applied at the beginning and end of the educative intervention (0 and 2 months), and 12 months afterwards. DM2 patients with CKD were evaluated during 1-year of follow-up with estimated glomerular filtration rate (eGFR) and albuminuria.

**Results:**

After educative intervention, there was a significant increase in FP clinical competence compared to baseline; although it was reduced after 1 year, it remained higher compared to baseline. One-hundred thirteen patients with early nephropathy (58 stage 1, 55 stage 2) and 28 with overt nephropathy (23 stage 3, 5 stage 4) were studied. At final evaluation, both groups maintained eGFR [(mean change) early 0.20 ± 19 pNS; overt 0.51 ± 13 mL/min pNS], whereas albuminuria/creatinuria (early −67 ± 155 *p* < 0.0001; overt −301 ± 596 mg/g *p* < 0.0001), systolic blood pressure (early −10 ± 18 *p* < 0.05; overt −8 ± 20 mmHg *p* < 0.05), and total cholesterol (early −11 ± 31 *p* < 0.05; overt −17 ± 38 mg/dL *p* < 0.05) decreased. Diastolic blood pressure, waist circumference and LDL-cholesterol were also controlled in early nephropathy patients.

**Conclusions:**

CPGs for Prevention, Diagnosis and Treatment of CKD, by means of an educative intervention increases FP clinical competence and improves renal function in DM2 patients with CKD.

## Introduction

Clinical practice guidelines (CPGs) are statements that include recommendations intended to optimize patient care; they are informed by a systematic review of evidence and an assessment of the benefits and harms of alternative care options (1). There is a large number of CPGs available in the area of Nephrology (2–8); however, there is no published evidence that the availability of such guidelines has reduced the inappropriate variation in practice or improved the clinical competence of physicians and, subsequently, the outcome of patients with chronic kidney disease (CKD). In fact, some data stress the important difficulties in achieving some guidelines targets in hemodialysis patients (9, 10). End-stage kidney disease (ESKD), mainly caused by type 2 diabetes mellitus (DM2), is an increasing public health problem in many parts of the world, particularly in our setting (11).

Educative models are among the best tools to counteract the ESKD epidemic; we have previously demonstrated that educative interventions increased clinical competence of health professionals and improve outcomes of DM2 patients with early CKD at primary healthcare units compared with not receiving educative interventions (12–14); however, the impact of CPGs implementation, as the central material of the intervention, has not been probed in these particular conditions.

Therefore, the aim of this study was to evaluate the effect of the implementation of CPGs for Prevention, Diagnosis and Treatment of Early CKD as the central educative material on the clinical competence of family physicians (FP) and kidney function of DM2 patients at a primary healthcare setting.

## Patients and methods

In Mexico, healthcare is provided by different systems: The Mexican Institute of Social Security (IMSS) is the major health provider covering ~51% of the total population (64 million persons) (15). In the state of Jalisco, the IMSS serves about 69.9% of the population, and in the city of Guadalajara, this institution has 20 primary healthcare units. In this prospective study, 3 out of the 20 units were randomly selected [Unidad de Medicina Familiar (UMF) No. 3, 51 and 52]. All FP working permanently on the diurnal shift of outpatient clinics were invited to participate; none of those who decided to enroll received salary compensation. Fifteen out of 25 FP from UMF No. 3, 15 out of 20 from UMF No. 51, and 7 out of 9 from UMF No. 52 were included; all of them received an educative intervention about diabetic nephropathy by means of an interactive theory-practice model for 2 months (starting at the beginning of this study, March 2017).

The educational strategy was led by 1 investigator (PMM) and was based on the *CPGs for Prevention, Diagnosis and Treatment of Early CKD* that were created focusing on the management of early CKD at the primary healthcare, edited by the National Center for Health Technology Excellence (CENETEC), an official decentralized organism of the Mexican Ministry of Health that concentrated all the CPGs of the country (16). These guidelines were developed from previously published international CKD guidelines, following principles and standards proposed by the USA Institute of Medicine (1), the Guidelines International Network (17) and the Appraisal of Guidelines, Research and Evaluation (AGREE II) instrument (18). They were freely available on internet.

The educational strategy included an analytical GPCs review (1 h) and discussion of real clinical cases (1.5 h), 2 days/week, for a total of 40 h. After the 2-month intervention, no additional training or reinforcement was performed. To measure clinical competence, a previously validated self-response questionnaire for diabetic nephropathy (19) was applied to all participant physicians at baseline, at the end of the educative intervention (2 months) and 12 months after finishing such intervention. This questionnaire was applied directly to all FP from each medical unit; 45 min was pre-established to complete the test. See *definitions* for more questionnaire details.

To determine the impact of possible changes in FP clinical competence, kidney function of their patients with DM2 and nephropathy was evaluated. Patients ≥18 years of age, any gender and DM2 vintage, and without previous CKD diagnosis were identified in a screening program in the primary healthcare units; those with early CKD were invited to participate and included after verbal informed consent. Patients who had <3 evaluations were eliminated of the study. DM2 was previously diagnosed in all patients according to the American Diabetes Association (20). At screening, all causes of transitory albuminuria were excluded. Microalbuminuria was evaluated with dipsticks (Micral-test II; Roche Diagnostics GmbH, Mannheim, Germany) in a first-void urine sample. Positive results were confirmed by immunoturbidimetry (Vitros 5600 Integrated System; Ortho Clinical Diagnostics, Rochester, MN) and adjusted to creatinine urinary excretion. All patients had an evaluation at the beginning of the study, and trimestral evaluations during a year. At baseline, a detailed clinical examination was performed, albuminuria/creatininuria ratio was quantified in a first morning urine sample, and a blood sample was obtained. In the latter, glycated hemoglobin (HbA_1C_), glucose, creatinine, and lipid levels were determined by using the usual methods. Glomerular filtration rate was estimated (eGFR) by using the Chronic Kidney Disease Epidemiology Collaboration (CKD-EPI) formula (21). The same clinical and biochemical evaluations performed at baseline were repeated every 3 months. All laboratory determinations were performed in the Central Laboratory of the Hospital de Especialidades, Centro Médico Nacional de Occidente, IMSS, by the same personnel blinded to patients' details. All patients had routine monthly visits and management according to their FP clinical criteria; investigators did not participate in treatment decisions. Regular visits of patients like these to an endocrinologist, cardiologist, or nephrologist are not part of the current practice in our setting unless specific problems are detected (i.e., ESKD or cardiovascular events). Patients in this study did not receive any educative intervention to self-manage DM2 or CKD. Medication and all issues related to healthcare are free in the IMSS system. The study was approved by the local Research and Ethics Committee (No. 2009/785/068).

### Definitions

Clinical competence was defined as the capability of FP to identify risk factors, integrate diagnosis, and correctly use laboratory tests and therapeutic resources in DM2 patients with nephropathy. For clinical competence measurement, a previously validated questionnaire (19) was used. This instrument consisted of 150 questions: 30 questions determine the ability to identify risk factors; 40, the ability for diagnostic integration; 40, the correct use of laboratory tests; and 40, the correct use of therapeutic resources. Levels of clinical competence were measured by using an ordinal scale: 0 to 23 successful answers, no better than random chance; 24–49 successful answers, very low; 50–75 successful answers, low; 76–100 successful answers, average; 101–125 successful answers, high; and 126–150 successful answers, very high. To identify different levels of clinical competence, the Pérez-Padilla and Viniegra formula (22) was used. To estimate the instrument reliability, the Kuder-Richardson coefficient was used as a measure of internal consistency (0.75).

CKD was classified according to the Kidney Disease: Improving Global Outcomes (KDIGO) guidelines (8). We defined early nephropathy as the presence of albuminuria (albumin/creatinine ratio, ≥30 mg/g) with normal eGFR or with the presence of mildly decreased eGFR (60 to 89 mL/min/1.73 m^2^). Overt nephropathy was defined as the presence of an eGFR <60 mL/min/1.73 m^2^.

Sample size was calculated for comparing paired differences in FP and patients, and considering information from previous study (12). A minimum increase of 18.5 correct answers in clinical competence of FP and a maintenance of eGFR ± 5 mL/min/1.73 m^2^ from baseline value in patients was considered appropriate; with 80% confidence level, alpha 0.05 and including 20% of possible losses, a sample size of 36 FP and 108 patients was finally calculated.

### Statistical analysis

Data are expressed as mean ± SD or median and 25th to 75th percentiles when dimensional variables had parametric or non-parametric distribution, respectively, or as percentage in the case of nominal variables. Intragroup analysis was performed using repeated measures ANOVA, repeated measures ANOVA on ranks, paired-samples Student *t*, Wilcoxon, or McNemar tests, as appropriate. A *p*-value <0.05 was accepted.

## Results

### Results on clinical competence of family physicians

Thirty-seven FP were included in the study. All of them received 100% of training sessions and had the baseline and post-intervention (2-month) evaluations; 31 physicians had also the final evaluation (12-month after the educative intervention). Age of FP was 41.5 ± 9.2 years, 59% were women, and their labor experience was 12.4 ± 7.4 years.

**Figure 1:**
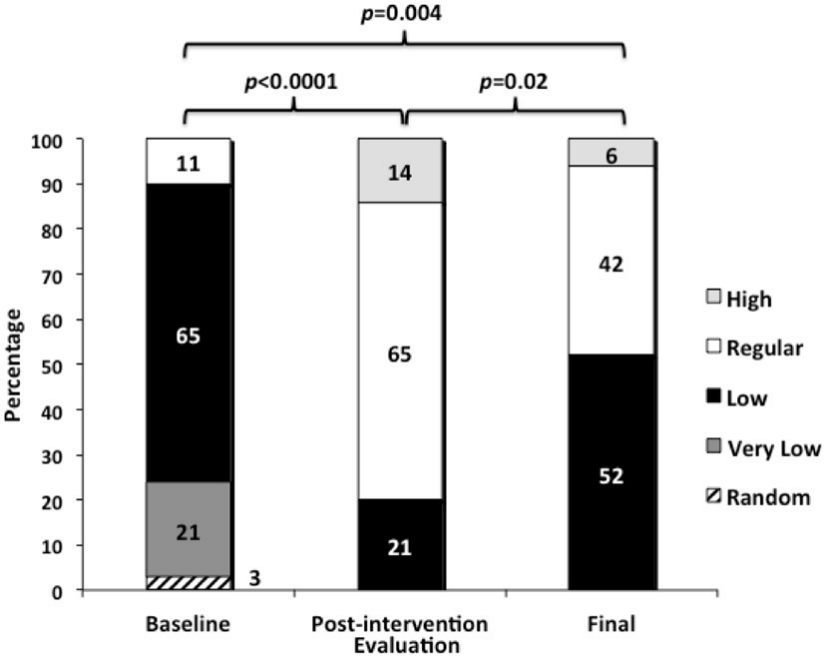
Comparisons of the FP clinical competence throughout the study.

Results of FP clinical competence are shown in Figure 1. At the baseline evaluation, most physicians had a low or very low level of clinical competence, only 10% had regular and none had high level; however, after the 2-month educative intervention, a significant improvement was observed as most of them reached a regular level and some reached the high level. A percentage of physicians that initially increased their competence displayed a reduction after 1 year of the intervention; however, their levels were still significantly higher compared to baseline.

Comparisons of clinical competence of FP by questionnaire's indicators are shown in Table 1. Immediately after the intervention, a significant increase in each of the indicators was observed. One year after the intervention, indicators such as diagnostic integration, correct use of laboratory tests and colleague's criticism decreased in comparison with the previous values, however, in general all the indicators were higher compared to baseline values.

**Table 1:** Comparison of clinical competence number of correct answers by indicator in family physicians throughout the study.

	**Baseline**	**Post-intervention**	**Final**
Risk factor identification	19.1 ± 3.7	21.6 ± 2.8^*^	22.0 ± 22.7^*^
Diagnostic integration	9.0 ± 7.7	21.6 ± 7.3^*^	15.3 ± 7.2^*^^£^
Correct use of laboratory tests	12.5 ± 4.4	15.5 ± 3.1^*^	13.3 ± 3.0
Correct use of therapeutic resources	9.7 ± 3.9	11.7 ± 3.0^*^	12.0 ± 3.5^*^
Colleague's criticism	13.6 ± 4.6	16.7 ± 6.0^*^	14.3 ± 4.9

* *p* < 0.05 vs. baseline evaluation.

£ *p* < 0.05 vs. post-intervention (2-month) evaluation.

### Results on kidney function of patients

A total of 660 DM2 patients were scrutinized; 165 had a confirmed CKD diagnosis but only 141 fulfilled the selection criteria, decided to participate, and completed the study: 113 patients with early nephropathy (58 stage 1, and 55 stage 2) and 28 with overt nephropathy (23 stage 3, and 5 stage 4).

At baseline, patients with overt nephropathy had significantly older age, lower frequency of male sex and tobacco consumption, higher frequency of hypertension, and longer duration of diabetes than those with early nephropathy (Table 2).

**Table 2:** Comparison of baseline sociodemographic characteristics and chronic complications of patients with early and overt nephropathy.

**Variable**	**Early nephropathy**	**Overt nephropathy**	***p*-value**
Number of patients	113	28	
Age (years)	63 ± 11	71 ± 8	0.0001
Male gender, *N* (%)	82 (73)	14 (50)	0.02
Elementary education, *N* (%)	77 (68)	19 (68)	0.88
Smoking, *N* (%)	22 (19)	1 (4)	0.04
Alcoholism, *N* (%)	30 (26)	5 (18)	0.35
Hypertension, *N* (%)	74 (65)	27 (96)	0.001
Duration of hypertension (years)	10 (4–15)	10 (5–15)	0.68
Duration of diabetes (years)	13 (8–18)	16 (11–20)	0.03
Cardiovascular disease, *N* (%)	23 (20)	5 (18)	0.73
Retinopathy, *N* (%)	67 (59)	19 (68)	0.43

In general, intermediate patients' evaluations (3 and 6 months) were in agreement with final results; henceforth, to simplify data presentation, only baseline and final results are shown. At the beginning of the study, it was noteworthy the presence of overweight-obesity and the inadequate control of serum glucose in both groups; with the latter being significantly worst in patients with early nephropathy (Table 3); adequate blood pressure, abdominal waist and lipid control was observed in the half or less of patients. At the end of the follow-up, patients with early CKD had a significant better control of systolic and diastolic blood pressure, LDL-cholesterol and triglycerides compared to baseline, whereas patients with overt CKD achieved the target for glycated hemoglobin significantly more frequently at final evaluation (Table 3). As expected by definitions, patients with overt nephropathy had significantly higher baseline serum creatinine and lower eGFR than their counterparts; however, both groups displayed an adequate preservation of eGFR and a significant decrease of albuminuria at the end of the study (Table 4).

**Table 3:** Comparison of achievement of recommendations of clinical and biochemical variables in patients with early and overt nephropathy.

**Variable**	**Target**	**Early nephropaty** **(*****N*** **113)**		**Overt nephropathy** **(*****N*** **28)**	
		**Baseline (%)**	**Final (%)**	**Baseline (%)**	**Final (%)**
Systolic BP (mmHg)	< 130	43	68^*^	38	48
Diastolic BP (mmHg)	< 80	28	41^*^	24	28
Body mass index (Kg/m^2^)	18.5–24.9	18	17	10	10
Waist circumference (cm)					
Males	≤ 102	53	54	60	47
Females	≤ 88	10	16	29	21
HbA_1C_ (%)	≤ 7	16^†^	32^*^^†^	31	51^*^
LDL-cholesterol (mg/dL)	< 100	36	41^*^	38	31
Triglycerides (mg/dL)	< 150	40	42	31	45

BP, blood pressure; HbA1C, glycated hemoglobin A_1*C*_; LDL, low density lipoprotein.

† *p* < 0.05 vs. overt nephropathy.

* *p* < 0.05 vs. baseline value of the same group.

**Table 4:** Comparison of kidney variables at baseline and final evaluations in patients with early and overt nephropathy.

**Variable**	**Early nephropaty** **(*****N*** **113)**		**Overt nephropathy** **(*****N*** **28)**	
	**Baseline**	**Final**	**Baseline**	**Final**
Serum creatinine (mg/dL)	0.80 (0.70–1.00)^†^	0.80 (0.68–1.02)	1.30 (1.20–1.85)^†^	1.40 (1.10–2.10)
Estimated GFR (mL/min/1.73 m^2^)	91.2 (74.6–109.1)^†^	89.1 (74.7–110.9)	47.6 (35.2–56.7)^†^	45.4 (32.8–59.1)
Albuminuria/creatininuria rate	121.9 (58.3–260.4)	56.5 (21.1–148.4)^*^	182.0 (43.6–537.5)	92.0 (22.7–397.3)^*^

GFR, glomerular filtration rate.

† *p* < 0.05 vs. overt nephropathy.

* *p* < 0.05 vs. baseline value of the same group.

Regarding the pharmacological treatment (Table 5), at baseline, patients with early nephropathy significantly used a lower number of antihiypertensive drugs, more angiotensin converting enzyme inhibitors (ACEIs) and less and angiotensin receptor blockers (ARBs) than those with overt nephropathy. At the final evaluation, both groups notably increased the number of antihypertensives, the use of ACEIs, ARBs, insulin+oral hypoglycemic drugs, statins, fibrates, and aspirin (at cardioprotective dose); however, in patients with overt nephropathy the change was significant only in the case of the first two variables.

**Table 5:** Comparisons of baseline and final treatment of patients with early and overt nephropathy.

**Variable**	**Early nephropathy** **(*****N*** **113)**		**Overt nephropathy** **(*****N*** **28)**	
	**Baseline**	**Final**	**Baseline**	**Final**
Number of antihypertensives	1.09 ± 0.88^†^	1.48 ± 0.86^*^	1.43 ± 0.96	2.04 ± 0.99^*^
ACEIs (%)	52^†^	75^*^	29	62^*^
ARBs (%)	15^†^	25^*^	29	42
Antidiabetics				
Insulin (%)	31	33	29	50
OHGs (%)	82	80	83	67
Insulin + OHGs (%)	19	27^*^	17	21
Hypolipemiants				
Statins (%)	32	46^*^	34	52
Fibrates (%)	21	31^*^	29	46
NSAIDs				
Aspirin (%)	34	49^*^	37	46
Other (%)	19	13	17	21

ACEIs, angiotensin converting enzyme inhibitors; ARBs, Angiotensin receptor blockers; OHGs, oral hypoglycemic drugs; NSAIDs, non-steroidal anti-inflammatory drugs.

† *p* < 0.05 vs. overt nephropathy.

* *p* < 0.05 vs. baseline value of the same group.

## Discussion

To the best of our knowledge, the present is the first study showing that an educative intervention based on CPGs implementation increased the clinical competence of FP and subsequently preserved eGFR, reduced albuminuria and improved other clinical and biochemical variables in DM2 patients with CKD. It is remarkable the virtually absent information about the impact of CPGs in the area of Nephrology; the very scarce information available suggest a lack of a positive effect. For instance, the COSMOS study (9) showed that a great percentage of hemodialysis patients across Europe were quite outside the K/DOQI and KDIGO recommended ranges of the main biochemical parameters for CKD–mineral and bone disorders. In other study, only 47% facilities of the Japanese cohort Dialysis Outcomes and Practice Pattern Study (J-DOPPS) adopted politics of treatment according to Institutional CPGs to control serum phosphorus according to the Guidelines for CKD-mineral and bone disorders in hemodialysis patients (10). In contrast to many Nephrology CPGs, the guidelines in which the present study was based on, were created essentially for early CKD and its adequate management at the primary healthcare, as one of the best option to fight against ESKD (23).

A rapid growth of CPGs has been observed in the last decades; they were intended to translate research into practice, to reduce practice variation, and to promote excellence in care. CPGs are fundamentals of the evidence-based movement, building the best available evidence on systematic reviews. Two key features in developing guidelines are to ensure that recommendations are based on high-quality systematic reviews of the best available evidence and that the review process to identify and grade relevant evidence is systematic, transparent and unbiased (24). CPGs used in the present educative intervention were focused on the management of early CKD at the primary healthcare setting. They were created according to international standards (1, 17, 18), and adapted to regional conditions. These guidelines were published on the website of the National Center for Health Technology Excellence (CENETEC) (16).

In healthcare, clinical guidelines are important instruments with which to improve and manage the care process (24). Dissemination and implementation are important issues for guidelines to promote compliance with recommended practices and to improve cost-effectiveness of interventions, ideally resulting in improved health outcomes (25). These strategies may use patient- or professional-mediated interventions, including educational outreach, distribution of educational materials, audit and feedback, among others; however, it is not known which strategy is the best to promote the introduction of guidelines into practice (25).

The educative intervention employed in the present study was based on an interactive model that has been previously shown as effective in the management of early CKD at the primary healthcare setting (12, 14), however, this was the first time that CPGs constitutes the main body of such educational intervention and it was shown to be effective. Clinical competence of FP significantly increased just after finishing the educative intervention, and although it decreased 1 year afterwards (time during which FP did not receive further training or reinforcement), it continued to be higher than before the intervention. The latter finding would suggest that cyclic or periodic educative interventions are necessary to keep competence of physicians at optimal levels. Interestingly, despite a trend to decrease clinical competence of FP at the end of the study, an adequate preservation of eGFR and a significant improvement of albuminuria, blood pressure, waist circumference and lipid control were achieved simultaneously with the more frequent use of nephroprotective drugs, which may further emphasize the positive impact of the educative intervention. These findings were more notable in the case of patients with early CKD, as previously shown (13); however, patients with overt CKD also received the benefit of an increased clinical competence of their physicians.

Policy makers need to have information about the likely benefits and costs of different guideline dissemination and implementation strategies if they are to make decisions about whether it is worthwhile to introduce guidelines. Unfortunately, there is a paucity of high-quality economic evaluations at the present time; this area deserves further investigation.

### Limitations and strengths of the study

The lack of a control group may be seen as a limitation of the study. However, the effectiveness of an educative intervention has been demonstrated previously by our team comparing *vs*. no intervention in a control group (12) or between different healthcare models (14); moreover, this issue was not the aim of the present study. Additionally, including a group of FP without a previously shown effective educative intervention could have some ethical implications, and the local ethics committee recommended to avoid this comparison. Educative interventions directed to patients have been also shown to improve negative lifestyles and have positive effects on kidney health (23) and on biomedical, behavioral, and psychosocial outcomes in patients with diabetes (26); patients in this study, however, did not receive any specific educative intervention in this regard. A possible bias could be that patients accepting and completing the study may have been those with higher interest or compliance to treatment. Other possible influencing variables such as health policy or medication availability were not modified during the time of this study.

Results could be perceived as biased by a relatively small sample size of medical units, physicians, and patients; however, simple size was calculated *a priori* as adequate using previous experiences of our research group.

In conclusion, CPGs for Prevention, Diagnosis and Treatment of CKD, implemented by means of an educative intervention positively influenced the clinical competence of FP, preserved eGFR, reduced albuminuria and improved other clinical and biochemical variables in DM2 patients with CKD at a primary healthcare setting.

## Data availability statement

The raw data supporting the conclusions of this article will be made available on reasonable request by the corresponding author.

## Ethics statement

The studies involving human participants were reviewed and approved by Comité Local de Investigación en Salud No. 1301. Written informed consent for participation was not required for this study in accordance with the national legislation and the institutional requirements.

## Author contributions

PM-M and AMC-M conceived the research idea and performed data analysis. PM-M, AMC-M, and HM-R made data curation and defined methodology of the protocol. PM-M and AMC-M wrote the manuscript with support of LC-S, HM-R, ER-C, and AH-H. All authors discussed the results and contributed to the final manuscript. All authors contributed to the article and approved the submitted version.
